# Indications for nerve-sparing surgery for radical prostatectomy: Results from a single-center study

**DOI:** 10.3389/fonc.2022.896033

**Published:** 2022-07-29

**Authors:** Zaisheng Zhu, Yiyi Zhu, Yunyuan Xiao, Shengye Hu

**Affiliations:** ^1^ Department of Urology, Jinhua Hospital Affiliated to Zhejiang University School of Medicine, Jinhua, China; ^2^ National Health Commission (NHC) Key Laboratory of Endocrinology, Department of Endocrinology, Peking Union Medical College Hospital, Peking Union Medical College, Chinese Academy of Medical Sciences, Beijing, China

**Keywords:** radical prostatectomy, prostate cancer, nerve-sparing, preoperative surgical indications, transperineal template-guided prostate biopsy, prostate MP-MRI examination

## Abstract

**Purpose:**

To explore the clinical indications of using the nerve-sparing technique in radical prostatectomy.

**Patients and methods:**

We retrospectively analyzed the clinical and pathological data of 101 patients who underwent radical prostatectomy (RP) at our institution. Twenty-five patients underwent open surgery, and 76 patients underwent laparoscopic surgery. The biochemical recurrence (BCR) rate was analyzed by the method of Kaplan–Meier. The distance between the ipsilateral neurovascular bundles (NVBs) and foci of prostate tumor (N-T distance) was measured in postoperative specimens. We defined the N-T distance >2 mm as the threshold to perform nerve-sparing (NS) in RP. Through logistic regression analysis, we determined the preoperative clinical indications for the nerve-sparing technique in RP.

**Results:**

The average BCR-free survival time was 53.2 months in these 101 patients with RP, with the 3- and 5-year BCR-free rates being 87.9% and 85.8%, respectively. The N-T distance was measured in 184 prostate sides from postoperative specimens of 101 patients. Univariate analysis showed that the percent of side-specific biopsy cores with cancer (≥1/3), maximum tumor length in biopsy core (≥5 mm), average percent involvement of each positive core (≥50%), PI-RADS score, and prostate MP-MRI imaging (extra-capsular extension) were associated with the N-T distance (*p* < 0.003). Furthermore, the percent of side-specific biopsy cores with cancer (≥1/3) (OR = 4.11, *p* = 0.0047) and prostate MP-MRI imaging (extra-capsular extension) (OR = 3.92, *p* = 0.0061) were found to be statistically significant independent predictors of the N-T distance in multivariate analysis.

**Conclusions:**

The clinical indications of nerve-sparing RP were <1/3 side-specific biopsy cores with cancer and no extra-capsular extension by prostate MP-MRI examination.

## Introduction

With the widespread use of prostate-specific antigen (PSA) screening, a majority of prostate cancers are diagnosed in the early stages (1,2). Combination with multiparametric magnetic resonance (MP-MRI) helps better estimate the prostate cancers and guides the treatment approaches (3). Radical prostatectomy (RP) is regarded as a standard treatment for localized prostate cancer. Although effective at cancer control, RP has several common complications including erectile and urinary dysfunction, which severely affects the quality of life. Therefore, the trifecta outcome (cancer control, urinary continence, potency recovery) has become the most desired outcome following RP. The nerve-sparing (NS) modification of RP is required for maintaining sexual functioning after surgery (4). The necessary surgical procedure of NS-RP is to maintain the integrity of the neurovascular bundle (NVB). The distance between the ipsilateral neurovascular bundles (NVBs) and foci of prostate tumor (the N-T distance) (5), measured in specimen removed by RP, is a practical way for determining indications that warrant the use of NS-RP. However, the N-T distance is a postoperative measurement tool, and we still need preoperative clinical indications. In this study, we propose the clinical indications of NS-RP and discuss the clinical safety and feasibility.

## Patients and methods

### Patient

We collected the data from patients with localized prostate cancer who performed RP at our institution from January 2015 to December 2019. The study included patients with intact clinical and pathological data after RP with at least one-side nerve excision. Moreover, all participants had more than a 1-year follow-up. The exclusion criteria were (I) lack of preoperative biopsy and MP-MRI image; (II) history of preoperative androgen blocking therapy; (III) performance of both-side NS-RP; (IV) less than 1 year of follow-up period; and (V) bone metastases through the radionuclide bone scan. Finally, 101 cases were collected and retrospectively analyzed. The biochemical recurrence (BCR) rate was defined as two consecutive PSA levels after RP >0.2 ng/ml. None of the patients received adjuvant therapy such as endocrine drugs and external radiotherapy after RP, unless in case of biochemical recurrence.

### Clinical data collection

Anthropometric measurements (age, weight, and height) were measured by trained nurses using a standardized protocol. Body mass index (BMI) was calculated by using the following formula: weight in kilograms divided by height in meters squared. The venous blood sample was taken in a fasting state at 8:00 a.m., and serum prostate-specific antigen was measured. Prostate Imaging–Reporting and Data System (PI-RADS) (6) was calculated based on MP-MRI prostate image, which was taken by a single radiologist specializing in the prostate. All the patients completed the International Prostate Symptom Score (IPSS) assessment questionnaire.

### Transperineal template-guided prostate biopsy

Transperineal template-guided prostate biopsy (TTPB) was completed before RP. Firstly, prostate volume was calculated using the prostate ellipsoid formula (width × length × height × π/6). The first biopsy was divided into two ways depending on prostate size. As for prostate volume of <50 cm^3^, 8+X cores were taken as designated by a standardized biopsy scheme, including the lateral (two symmetrical needles respectively), the junctional region between the medial and lateral (one symmetrical needle respectively), the medial (one symmetrical needle respectively), and the suspected region, like the abnormal prostate nodule (X symmetrical needles). As for prostate volume of >50 cm^3^, 10+X or 12+X cores were taken. Based on 8+X, the 10+X scheme added two more symmetrical needles in the junctional region between the medial and lateral. Moreover, the 12+X scheme added two more symmetrical needles in the apex, based on the scheme of 10+X. Moreover, the repeated prostate biopsy was adopted in men with persistent suspicion of prostate tumor after a negative initial biopsy. The location of repeated prostate biopsy included the lateral (three symmetrical needles respectively), the junctional region between the medial and lateral (three symmetrical needles respectively), the medial (two symmetrical needles respectively), and the apex (two symmetrical needles respectively). The data including the percent of side-specific biopsy cores with cancer, maximum tumor length in biopsy core, and average percent involvement of each positive core were acquired from TTPB. The biopsy Gleason score was calculated and graded by samples from prostate biopsies.

### Pathological characteristics after radical prostatectomy

**Figure 1:**
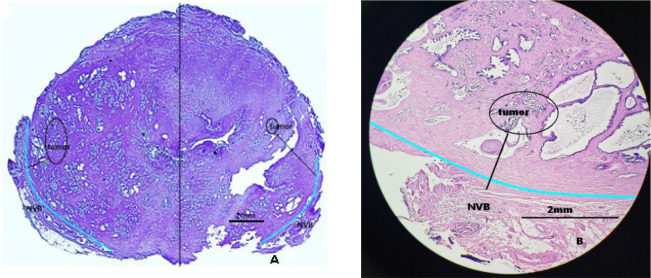
Schematic diagram of distance between the neurovascular bundle (NVB) and the foci of prostate cancer. **(A)** The measurement method of distance between the neurovascular bundle and the foci of prostate cancer: performed separately on the left and right sides. The light blue line is the prostate capsule (the junction of the gland and NVB). **(B)** The specimen measured the distance between the neurovascular bundle and the foci of prostate cancer.

The pathological specimens of the prostate were sectioned at intervals of 3–4 mm and were stained with hematoxylin and eosin for morphological evaluation. Optical microscopy (OM) was used to observe microstructures of specimens and measure the N-T distance (**Figure 1**). NS-RP was permitted on the condition of the N-T distance >2 mm, while it was considered as a contraindication when the N-T distance ≤2 mm.

### Statistical analyses

Univariate and multivariate logistic regression analyses were conducted for the N-T distance (≤2 or >2 mm) associations with independent variables. The independent variables included preoperative PSA level, prostate volume, Gleason score, tumor risk classification, percent of side-specific biopsy cores with cancer, maximum tumor length in biopsy core, average percent involvement of each positive core, PI-RADS score, and extra-capsular extension in MP-MRI image. BCR-free survival was calculated with the Kaplan–Meier method. The SPSS ver. 25 (SPSS Inc., Chicago) was used for statistical analysis. *p* < 0.05 was considered to indicate statistical significance.

## Results

### Preoperative clinical characteristics

A total of 101 patients were enrolled according to the study criteria. The basal clinical manifestations are summarized in **Table 1**. The median age, body mass index (BMI), IPSS score, preoperative PSA level, and prostate volume were 66.3 (51–78) years, 25.36 (21–31) kg/m^2^, 6 (0–19), 8.96 (3.4–36.6) ng/ml, and 41.32 (19–87) ml, respectively. No obvious lymph node metastasis was observed in the preoperative MP-MRI image, while 15 patients (14.85%) had extra-capsular extension. Using PI-RADS (6), 13 patients (12.87%), 51 patients (50.50%), 23 patients (22.77%), and 14 patients (13.86%) were scored 1–2, 3, 4, and 5, respectively. Using the clinical TMN staging system, 68 (67.33%) patients were in the cT1 stage, 18 (17.82%) patients were in the cT2 stage, and 15 (14.85%) patients were in the cT3 stage. TTPB showed that the median needle was 9.38 (8–20), including 29.14% (276/947) being a positive needle. Thirty-two patients (26.73%) had side-specific biopsy cores with cancer ≥1/3. The median diameter of the pathological specimen was 4.13 mm (0.5–15), and 27 patients (26.73%) had maximum tumor length in biopsy core ≥5 mm. Twenty-five patients (24.95%) had average percent involvement of each positive core ≥50%. The median biopsy Gleason score was 6.72 (6–9), consisting of six in 39 patients (38.61%), seven in 52 patients (51.49%), and eight–nine in 10 patients (9.90%). Based on serum PSA, biopsy Gleason score, and clinical stage, 29 patients (28.71%), 39 patients (38.61%), and 13 patients (12.87%) were divided into the low-risk group, medium-risk group, and high-risk group, respectively.

**Table 1:** Baseline characteristics of the study population.

Characteristics	Patients (*n* = 101)
Age (years)	66.30 (51-78)
BMI (kg/m^2^)	25.36 (21-31)
IPSS score	6 (0-19)
Preoperative PSA (ng/mL)	8.96 (3.4-36.6)
Prostate volume (mL)	41.32 (19-87)
Clinical T stage	
cT1	68 (67.33%)
cT2	18 (17.82%)
cT3	15 (14.85%)
Biopsy Gleason score	
6	39 (38.61%)
7	52 (51.49%)
8-9	10 (9.9%)
D’Amico risk classification	
Low	29 (27.71%)
Intermediate	39 (38.61%)
High	13 (12.87%)
PI-RADS	
1-2	13 (12.87%)
3	51 (50.50%)
4	23 (22.77%)
5	14 (13.86%)
MP-MRI image (capsular invasion)	15 (14.85%)
Variables with preoperative needle biopsy	
Numbers of biopsy cores	9.38 (8-20)
Percent of side-specific cores with cancer (≥1/3)	32 (31.68%)
Maximum tumor length in biopsy core (mm)	4.13 (0.5-15)
Maximum tumor length in biopsy core (≥5 mm)	27 (26.73%)
Average percent involvement of each positive core (≥50%)	25 (24.75%)
Operation	
Open surgery	25 (24.75%)
Laparoscopic surgery	76 (75.25%)

BMI, body mass index; IPSS, International Prostate Symptom Score; PSA, prostate-specific antigen; PI-RADS, Prostate Imaging–Reporting and Data System; MP-MRI, multiparameter MRI.

### Surgical methods and pathological specimen characteristics

**Figure 2:**
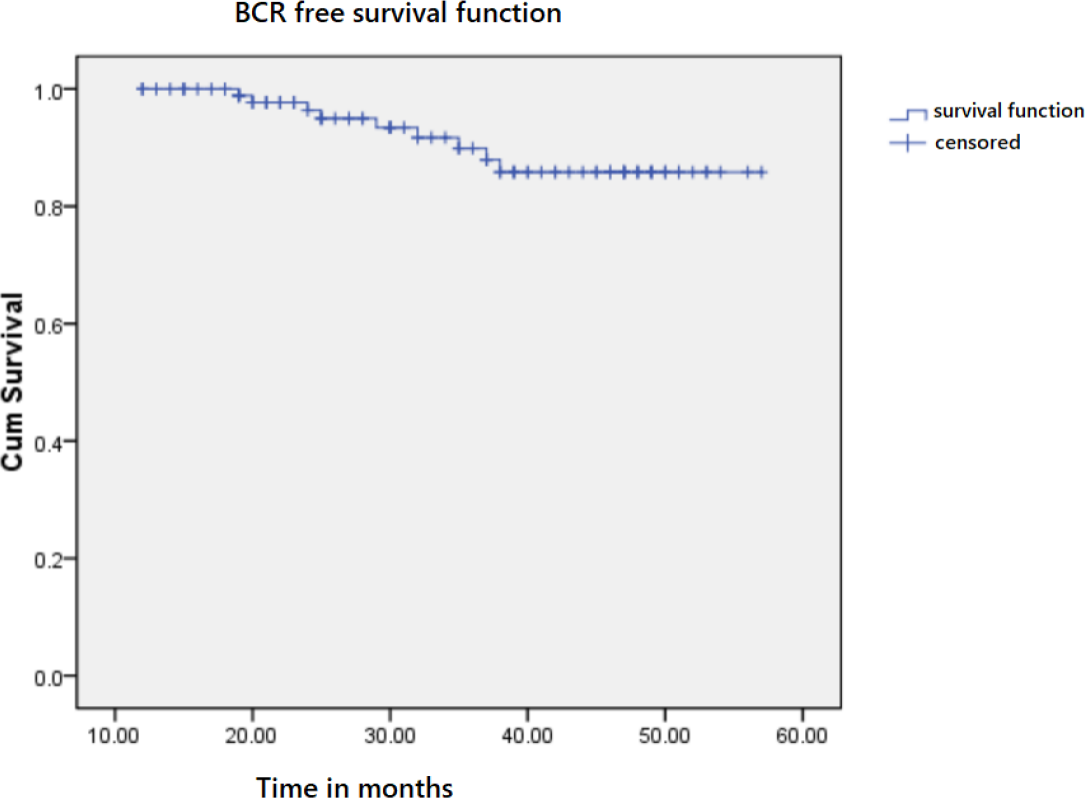
Kaplan–Meier curve for overall BCR-free survival time.

Of all the 101 patients, open surgery was done for 25 (25.75%) and laparoscopic surgery for 76 (75.25%). Bilateral non-NVB preservation was done for 83 (82.18%), while unilateral was done for 18 (17.82%). According to postoperative pathological stage, 17 (16.83%) patients were in stage ≤pT2b, 59 (58.42%) patients in stage pT2c, 18 (17.82%) in stage pT3a, and 7(6.93%) in stage pT3b. A positive surgery margin (PSM) was observed in nine patients (8.91%), with four cases at the base, four cases at the posterior, and one case at the apex. All 101 patients experienced more than 1 year of the follow-up visit. The median BCR survival time was 53.2 (50.87–55.52) months. The 3- and 5-year BCR-free survival rates were 87.9% and 85.8%, respectively (**Figure 2**).

### Logistic regression analyses of the N-T distance

The N-T distance was measured in 184 prostate sides from postoperative specimens of 101 patients, with 68 sides (36.96%) ≤2 mm and 116 sides (63.04%) >2 mm. There were significant differences in PI-RADS score (≥4), extra-capsular extension in MP-MRI image, and preoperative needle biopsy-related factors, including percent of side-specific biopsy cores with cancer (≥1/3), maximum tumor length in biopsy core (≥5 mm), and average percent involvement of each positive core (≥50%) (*p* < 0.003, **Table 2**). No differences were shown in age, BMI, IPSS score, PSA level, prostate volume, biopsy Gleason scores, and tumor risk classification (*p* > 0.05). In the multivariate analysis, extra-capsular extension in MP-MRI image (OR = 3.92, *p* = 0.0061) and percent of side-specific biopsy cores with cancer (≥1/3) (OR = 4.11, *p* = 0.0047) were identified as independent risk factors associated with the N-T distance (**Table 3**).

**Table 2:** Univariate analysis of preoperative variances and the N-T distance.

Variables	Distance ≤2 mm (68 sides/%)		Distance >2 mm (116 sides/%)	χ^2^	*p*
**Preoperative clinical variables**					
Age (≥70 year)		23 (33.82)	41 (35.34)	0.044	0.834
BMI (≥28 kg/m^2^)		11 (16.18)	14 (12.07)	0.616	0.433
IPSS score (≥9)		7 (10.29)	17 (14.66)	0.719	0.397
PSA (≥10 ng/ml)		22 (32.35)	33 (28.45)	0.312	0.577
Prostate volume (≥50 ml)		10 (14.71)	16 (13.79)	0.029	0.864
Biopsy Gleason score (≥7)		25 (36.76)	30 (25.86)	2.432	0.119
Risk classification (high-risk)		9 (13.24)	19 (16.38)	0.328	0.567
PI-RADS (≥4)		33 (48.53)	26 (22.41)	13.422	0.000
MP-MRI image (extra-capsular extension)		12 (17.65)	5 (4.31)	9.093	0.003
**Variables with preoperative needle biopsy**					
Percent of side-specific biopsy cores with cancer (≥1/3)		37 (54.41)	18 (15.52)	30.946	0.000
Maximum tumor length in biopsy core (≥5 mm)		23 (33.82)	14 (12.07)	12.629	0.000
Average percent involvement of each positive core (≥50%)		19 (27.94)	7 (5.88)	16.955	0.000

BMI, body mass index; PSA, prostate-specific antigen; IPSS, International Prostate Symptom Score; PI-RADS, Prostate Imaging–Reporting and Data System; MP-MRI, multiparameter MRI.

**Table 3:** Multivariate analysis of preoperative variances and the N-T distance.

Variable		OR (95% CI)	*p*
**Preoperative clinical variables**			
Age (≥70 years)	1.02 (0.39-3.47)		0.633
BMI (≥28 kg/m^2^)	0.69 (0.31-2.98)		0.542
IPSS score (≥9)	0.71 (0.46-3.86)		0.527
PSA (≥10 ng/ml)	0.99 (0.39-2.21)		0.984
Prostate volume (≥50 ml)	1.13 (0.42-2.13)		0.890
Biopsy Gleason score (≥7)	1.72 (0.33-3.06)		0.764
Risk classification (high-risk)	0.67 (0.10-1.78)		0.266
PI-RADS (≥4)	1.03 (0.33-9.97)		0.109
MP-MRI image (extra-capsular extension)	3.92 (1.49-10.03)		0.006
**Variables with preoperative needle biopsy**			
Percent of side-specific biopsy cores with cancer (≥1/3)	4.11 (1.56-9.51)		0.005
Maximum tumor length in biopsy core (≥5 mm)	1.09 (0.22-4.19)		0.872
Average percent involvement of each positive core (≥50%)	2.93 (0.44-19.12)		0.214

BMI, body mass index; PSA, prostate-specific antigen; IPSS, International Prostate Symptom Score; PI-RADS, Prostate Imaging–Reporting and Data System; MP-MRI, multiparameter MRI.

## Discussion

Radical prostatectomy (RP) is the main surgical treatment for localized prostate cancer, and nerve-sparing (NS) is recognized as a critical surgical procedure to preserve or restore erectile function. NS-RP improves sexual function and restores urinary continence, but it might increase the positive rate of the surgical margin (7,8). Until now, the indication of NS-RP remains ambiguous.

Previous studies suggested that the intraoperative visual and tactile examination of epfascial sclerosis nodule could be the determination on whether to spare the nerve in RP or not (9). However, due to open surgery being substituted by laparoscopic surgery, depending on intraoperative examination was not practical and precise. Furthermore, in some studies (5,10–12), the extra-prostatic extension (EPE) in the region of NVB was regarded as one of the most important clinical variants for NS-RP or not. Naya et al. (10) reported that the strongest preoperative independent predictors of the EPE were the maximum tumor length ≥7 mm and positive basal core location. Tsuzuki et al. (11) showed that PSA (>10 ng/ml), biopsy Gleason score (>7), average percent involvement of each positive core (>20%), percent of side-specific biopsy cores with tumor (≥1/3), and abnormalities in the rectal examination were statistically significant independent predictors of EPE in the NVB. However, rather than adopting the EPE in the NVB as a criterion, the N-T distance (>2 mm) in postoperative pathologic specimens was used in our study.

We suggested the N-T distance (>2 mm) as the criterion based on the following reasons: (I) the N-T distance is not only associated with PSM but also a more objective indicator, compared with the extra-prostatic extension. Therefore, using N-T distance as the criterion to evaluate NS is more consistent with the clinical principles of cancer control. (II) The network of nerve fibers and extra-capsular tumor cells are both microscopic, thus their anatomical structures are hard to be recognized visually. Despite using the three-dimensional DaVinci system, which magnifies the field by 10–12 times, their anatomical structures are still difficult to visualize intraoperatively, leading to more possibility of PSM. As a result, to keep the balance between tumor control and nerve-sparing, using the N-T distance from the postoperative specimen is a more objective indicator. (III) Sung et al. (13) demonstrated that the average distance from the prostatic capsule to nerve fibers was 1.73 mm in the ventral part, 1.80 mm in the dorsal (rectal) part, 1.58 mm in the right lateral part, and 1.23 mm in the left lateral part (*p* = 0.266). Also, no significant difference was observed among the base (1.62 mm), mid-part (1.41 mm), and apex levels (1.72 mm) (*p* = 0.673). Inoue et al. (5) reported that the mean N-T distance with EPE was 2.01, 1.95, and 1.85 mm in the apex, middle, and base, respectively. Therefore, PSM was very likely to occur when performing NS-RP when the N-T distance was <2 mm. (IV) In our previous study related to the network of nerve fibers under endoscopic technique, the network was divided into two groups and five zones. The most vulnerable areas were the peri-seminal vesicle reticulum (zone 1), NVB (zone 2), and bilateral prostatic network (zone 3). Through autopsy, we found that the nerve network in the ventral 12-point region of the pelvic fascia around the prostate was sparsely distributed and other regions were equally distributed. Hence, measuring the N-T distance in the NVB region as the criterion was anatomically appropriate. (V) Previous research showed that EPE happened in the NVB region and easily caused PSM (14,15). In our study, the percent of PSM happened in the NVB region and the base of the prostate was 89% (8/9). Thus, regarding the N-T distance as the criterion was consistent with the pathophysiology of prostate cancer.

Previous studies found that the EPE was associated with the percent of side-specific biopsy cores with tumor (≥1/3), preoperative PSA level, and biopsy Gleason score (5,14–17). Preoperative MP-MRI (18,19) is also related to EPE and is used to estimate the risk category of prostate cancer (3,20). Our study used the N-T distance (2 mm) as the criterion for NS-RP and predicted its associated preoperative clinical variants. The result showed that only the percent of side-specific biopsy cores with tumor (≥1/3) and preoperative MP-MRI examination were the independent risk variances. Based on the above results, we suggested that the preoperative indication of NS-RP was percent of side-specific biopsy cores with tumor (<1/3) and no extra-capsular extension by MP-MRI examination, even though PSA level >10 ng/ml or biopsy Gleason score >8 in clinical T2c cases.

Our study still had some limitations. The samples were relatively small due to exclusion of cases undergoing bilateral NS surgery, leading to selective deviation. For those bilateral NS cases, lacking neural markers resulted in the unreliable measurement of the N-T distance. Instead, we measured the distance between the posterolateral region of the prostate and tumor (<2 mm) as substitution and finally got similar results. We must point out that it remains to be further demonstrated the rationality of adopting the N-T distance (>2 mm) as the NS-RP criterion. Furthermore, more prospective studies should be designed to evaluate the above preoperative factors as the criterion of the NS-RP.

## Conclusion

For the patients with radical prostatectomy, percent of side-specific biopsy cores with tumor (<1/3) and no extra-capsular extension by MP-MRI examination can be regarded as preoperative indications for nerve-sparing surgery when we defined the distance between a tumor and neurovascular bundle >2 mm as the nerve-sparing criterion.

## Data availability statement

The original contributions presented in the study are included in the article/supplementary material. Further inquiries can be directed to the corresponding author.

## Ethics statement

The studies involving human participants were reviewed and approved by the ethics committee of Jinhua Hospital. The patients/participants provided their written informed consent to participate in this study. Written informed consent was obtained from the individual(s) for the publication of any potentially identifiable images or data included in this article.

## Author contributions

ZZ: study design, collecting of clinical data, and writing of the article. YZ: patient follow-up and collecting of clinical data. YX and SH: conducting of the statistical analysis. All authors contributed to the article and approved the submitted version.

## Funding

This work was supported by grants from the Basic Public Welfare Research Project of Zhejiang (LGF18H050006) and the Major Research Program of Jinhua Science and Technology (2021–3–022).

## Conflict of interest

The authors declare that the research was conducted in the absence of any commercial or financial relationships that could be construed as a potential conflict of interest.

## Publisher’s note

All claims expressed in this article are solely those of the authors and do not necessarily represent those of their affiliated organizations, or those of the publisher, the editors and the reviewers. Any product that may be evaluated in this article, or claim that may be made by its manufacturer, is not guaranteed or endorsed by the publisher.

## References

[1] Zaisheng Z Hongqi S Pengfei Z Yibo Z Chunting Z Qiang F. Application of pubovesical complex preserving technique during laparoscopic radical prostatectomy. Chin J Urol (2018) 39(7):515–21. doi: 10.3760/cma.j.issn.1000-6702.2018.07.009

[2] Inoue S Hieda K Hayashi T Teishima J Matsubara A. Longitudinal analysis of trifecta outcome in Japanese patients with prostate cancer following robot-assisted laparoscopic radical prostatectomy. World J Urol (2020) 40(8):2009–15. doi: 10.1007/s00345-020-03515-2 33185707

[3] Gentile F La Civita E Della Ventura B Ferro M Cennamo M Bruzzese D. A combinatorial neural network analysis reveals a synergistic behaviour of multiparametric magnetic resonance and prostate health index in the identification of clinically significant prostate cancer. Clin Genitourin Cancer (2022) S1558-7673(22)00091. doi: 10.1016/j.clgc.2022.04.013 35610113

[4] Morozov A Barret E Veneziano D Grigoryan V Salomon G Fokin I. A systematic review of nerve-sparing surgery for high-risk prostate cancer. Minerva Urol Nephrol (2021) 73(3):283–91. doi: 10.23736/S2724-6051.20.04178-8 33439578

[5] Inoue S Shiina H Hiraoka T Mitsui Y Sumura M Urakami S. Retrospective analysis of the distance between the neurovascular bundle and prostate cancer foci in radical prostatectomy specimens: its clinical implication in nerve-sparing surgery. BJU Int (2009) 104(8):1085–90. doi: 10.1111/j.1464-410X.2009.08592.x 19388988

[6] Zhipeng Z Ming L Min C Chunmei L Xin W Xuan W. Risk factors of prostate cancer in men with PI-RADS 1-2 lesions by multiparametric MRI. Chin J Urol (2021) 42(1):23–7. doi: 10.3760/cma.j.cn112330-20200119-00039

[7] Sammon JD Sharma P Trinh QD Ghani KR Sukumar S Menon M. Predictors of immediate continence following robot-assisted radical prostatectomy. J Endourol (2013) 27(4):442–6. doi: 10.1089/end.2012.0312 23030798

[8] Tewari AK Ali A Metgud S Theckumparampil N Srivastava A Khani F. Functional outcomes following robotic prostatectomy using athermal, traction free risk-stratified grades of nerve sparing. World J Urol (2013) 31(3):471–80. doi: 10.1007/s00345-012-1018-7 23354288

[9] Magheli A Burnett AL. Erectile dysfunction following prostatectomy: prevention and treatment. Nat Rev Urol (2009) 6(8):415–27. doi: 10.1038/nrurol.2009.126 19657376

[10] Naya Y Slaton JW Troncoso P Okihara K Babaian RJ. Tumor length and location of cancer on biopsy predict for side specific extraprostatic cancer extension. J Urol (2004) 171(3):1093–7. doi: 10.1097/01.ju.0000103929.91486.29 14767278

[11] Tsuzuki T Hernandez DJ Aydin H Trock B Walsh PC Epstein JI. Prediction of extraprostatic extension in the neurovascular bundle based on prostate needle biopsy pathology, serum prostate specific antigen and digital rectal examination. J Urol (2005) 173(2):450–3. doi: 10.1097/01.ju.0000151370.82099.1a 15643200

[12] Zaisheng Z Hongqi S Pengfei Z Yibo Z Lizhen X Yiyi Z. On individualized nerve retention during laparoscopic radical prostatectomy. Chin J Min Inv Surg (2020) 20(04):309–13. doi: 10.13263/j.cnki.nja.2020.02.015

[13] Sung W Lee S Park YK Chang SG. Neuroanatomical study of periprostatic nerve distributions using human cadaver prostate. J Korean Med Sci (2010) 25(4):608–12. doi: 10.3346/jkms.2010.25.4.608 PMC2844592 20358006

[14] Pavlovich CP Rocco B Druskin SC Davis JW. Urinary continence recovery after radical prostatectomy - anatomical/reconstructive and nerve-sparing techniques to improve outcomes. BJU Int (2017) 120(2):185–96. doi: 10.1111/bju.13852 28319318

[15] Hashimoto K Hisasue S Masumori N Kobayashi K Kato R Fukuta F. Clinical safety and feasibility of a newly developed, simple algorithm for decision-making on neurovascular bundle preservation in radical prostatectomy. Jpn J Clin Oncol (2010) 40(4):343–8. doi: 10.1093/jjco/hyp157 19959505

[16] Song W Park JH Jeon HG Jeong BC Seo SI Jeon SS. Comparison of oncologic outcomes and complications according to surgical approach to radical prostatectomy: Special focus on the perineal approach. Clin Genitourin Cancer (2017) 15(4):e645–e52. doi: 10.1016/j.clgc.2017.01.015 28216277

[17] Kaye DR Hyndman ME Segal RL Mettee LZ Trock BJ Feng Z. Urinary outcomes are significantly affected by nerve sparing quality during radical prostatectomy. Urology (2013) 82(6):1348–53. doi: 10.1016/j.urology.2013.06.067 24094657

[18] McClure TD Margolis DJ Reiter RE Sayre JW Thomas MA Nagarajan R. Use of MR imaging to determine preservation of the neurovascular bundles at robotic-assisted laparoscopic prostatectomy. Radiology (2012) 262(3):874–83. doi: 10.1148/radiol.11103504 22274837

[19] Thompson J Lawrentschuk N Frydenberg M Thompson L Stricker P Usanz. The role of magnetic resonance imaging in the diagnosis and management of prostate cancer. BJU Int (2013) 112 Suppl 2:6–20. doi: 10.1111/bju.12381 24127671

[20] Russo F Manfredi M Panebianco V Armando E De Luca S Mazzetti S. Radiological wheeler staging system: a retrospective cohort analysis to improve the local staging of prostate cancer with multiparametric MRI. Minerva Urol Nefrol (2019) 71(3):264–72. doi: 10.23736/S0393-2249.19.03248-X 30654601

